# 25 years of military load carriage

**DOI:** 10.1113/EP092409

**Published:** 2025-04-03

**Authors:** Christopher A. J. Vine, Nicholas Schofield

**Affiliations:** ^1^ Occupational Performance Research Group, Institute of Applied Sciences University of Chichester Chichester UK

We read with much interest the latest ‘Physiology of Lived Experiences’ editorial by Professor Tipton (Tipton, 2025), whereby he uses his own experiences to demonstrate the interdisciplinary nature of endurance events, especially as I (C.A.J.V.) had been taught by Professor Tipton at the University of Portsmouth and was therefore already aware of his 5‐yearly ‘heath check’. Given the novelty of this approach to translating ‘science to practice’, we were interested in discussing load carriage and the physiological and physical implications of our lived experiences. Although I am someone who enjoys hiking and have started to undertake multiday self‐supported hikes, the real focus of this piece will be those lived experiences of Nick (N.S.), who was in the British Army for 25 years within the Airborne Artillery, 16 Air Assault Brigade; a unit with load carriage heritage linking back to World War II in particular. An interesting parallel was that quantifying the physical demands and characteristics of the Parachute Regiment's ‘10‐miler’ (a 10‐mile load carriage event) was one of my first field data collection experiences as a postgraduate research assistant working with the British Armed Forces. Although this piece will focus on the lived experiences of load carriage, for a more in‐depth physiological review of load carriage, we refer the reader to Faghy et al. (2022).

In the occupational physiology sphere, load carriage refers to the action of moving via walking or running, whilst carrying an external load; typically, of mission‐specific equipment (Vine, Coakley, Blacker, Runswick et al., 2022), although there may also be periods when individuals are largely stationary with this load (e.g., checkpoints, vital asset protection). Depending on both the individual's occupation (e.g., soldier, firefighter, police officer) and their specific role (e.g., radio operator, medic), the load mass carried can vary substantially. For example, in general duty police officers, external load mass in the form of a duty belt and body armour can total ∼10 kg (Ehnes et al., 2020), whereas the representative patrol order used by the British Army for their physical employment standards is 40 kg (Rue et al., 2024). Critically, in soldiers this load can easily exceed 60% of an individual's body mass during times of operational need or within specialist roles, where additional equipment is required (e.g., battlefield radios, specialist weapon systems; Nindl et al., 2013; Orr, 2010). For example, in a report by Dean (2003) on external loads carried by US soldiers in Afghanistan, average regiment emergency approach march load was ∼60 kg, with some roles carrying ∼68 kg, or ∼97% of their body mass. Unsurprisingly, it is well documented that as load increases so do the metabolic requirements of the task (Faghy et al., 2022). For example, we measured the metabolic cost of carrying external load masses ranging between 25 and 70 kg (in a combination of belt webbing systems, day sack/bergan, weapon and body armour) at 2.5, 4.8 and 5.5 km h^−1^ in UK Ground Close Combat personnel and demonstrated ∼1% increase in metabolic cost for every additional kilogram of external load mass carried (Vine, Coakley, Blacker, Doherty et al., 2022). Importantly, I, and other recreational hikers, have the luxury of buying and selecting lighter clothing and equipment, whereas Nick's challenge throughout his career was balancing the trade‐off between reducing his external load and meeting operational requirements, all within the confines of the Ministry of Defence's approved equipment. The remainder of the editorial will be written from Nick's perspective.

## 1 THE EVOLUTION OF LOAD CARRIAGE ASSESSMENT

When I joined the military in 1993, the load carriage assessment was designed around the loads and distances covered in past military conflicts. The Falklands War had highlighted that some soldiers still struggled with load carriage over longer distances. My first load carriage assessment was 8 miles carrying 15 kg, which was to be completed in 1 h and 50 min. This assessment was completed every 6 months once I passed both basic and initial trade training. In comparison, at the time, Parachute Regiment soldiers (which I had not served with at the time) were required to complete 10 miles in the same time carrying 15 kg. This assessment was only completed on Pre‐Parachute selection but was used infrequently during battalion physical training. Throughout my time within the British Army, the load carriage assessment has evolved, with support from human performance experts, to the assessment we see today (see Table 1). This evolution aimed to reflect the physical requirements for each role group, supporting the British Army's change in military tactics and reflecting physical employment standards best practice (Reilly et al., 2019). A major intended downstream effect was that of reducing musculoskeletal injuries in soldiers. For some physiological context on these assessments, work rates have been estimated with the widely used Pandolf equation (Pandolf et al., 1977). However, for simplicity, adjustments for load mass carried in the hands (i.e., weapon) or the feet (i.e., combat boots) have not been made (Soule & Goldman, 1969).

**TABLE 1:** Descriptions of loads carried and the estimated physical demands of load carriage assessments completed by Nick during his time within the British Army.

Assessment	External load mass (kg)	Distance covered (km)	Duration (h:min:s)	Equipment ensemble	Body mass at time of completion (kg)	Estimated metabolic rate (W)^b^	Estimated relative metabolic rate (mL kg^−1^ min^−1^)	Estimated absolute metabolic rate (L min^−1^)
Combat Fitness Test (CFT)	15	12.87 (8 miles)	1:50:00	Webbing, day sack, body armour, helmet, weapon	57	577	29.0	1.65
Basic Combat Fitness Test (BCFT)	25*	12.87 (8 miles)	1:50:00	Bergan (with helmet inside), body armour, weapon	68	666	36.8	2.50
Role Fitness Test, Part 1	40	4	0:35:00^a^	Webbing, bergan, body armour, helmet, weapon	73	904	35.4	2.58
Role Fitness Test, Part 2	25	2	0:12:30^a^	Webbing, day sack, body armour, helmet, weapon	73	1315	51.5	3.76

*Note*: Estimated metabolic rate was calculated using the Pandolf equation (Pandolf et al., 1977), with gradient set to 1% and a terrain factor of 1.0 (tarmac) used alongside body mass at the time of completion. Data are converted from watts to millilitres per kilogram per minute using the equation described by Drain et al. (2017).

^a^ *Note* that these are specific fitness conditions for the Parachute Regiment and differ from standards for other parts of the British Army.

^b^ Limitations of this equation have been noted within the literature (e.g., Drain et al., 2017; Vine, Coakley, Blacker, Doherty et al., 2022).

## 2 BODY MASS AND COMPOSITION

When I joined the British Army as a Combat Infantryman in 1993, I weighed 57 kg and was 1.68 m tall. Initially, I was told I was underweight, but if I passed the physical assessment, I would be allowed to start training. Owing to my small stature, I was a good runner and could therefore easily pass the physical entry assessments consisting of generic gym‐based fitness tests. At the time, this was a 1.5 mile run, which I completed in just under 9 min (the pass standard was 10 min 30 s). Despite this level of fitness, when I first started to undertake load carriage activities in basic training, it was clear that my small stature would cause me problems when undertaking this role‐critical task. This realization materialized during both basic training and the Combat Infantryman's Course, where I struggled during load carriage tasks. In context, we were carrying ∼20 kg of external load during load carriage assessments, which was ∼35% of my bodyweight, whereas during field exercises we were carrying ∼40–50 kg, which equated to ∼70%–87% of my bodyweight. Despite this large physical burden, I managed to pass the Combat Infantryman's Course, and I joined my infantry battalion. It was, however, clear that my weight and physical performance during load carriage needed addressing. On reflection, I attribute my relatively low body mass as the main factor for me struggling with load carriage. Carrying a significant percentage of my body mass during load carriage impaired my ability to move my lower limbs quickly, thereby reducing my performance. This was further exacerbated when there was a requirement to run/jog with load, and I always found myself falling back during these periods. It was always challenging work then to catch back up with the squad, requiring me to expend further energy and develop further fatigue.

I started resistance training, and over 4 years I managed to gain ∼11 kg in body mass (weighing ∼68 kg in 1997). With this increase in mass came a dramatic improvement in physical performance, which allowed me to complete more arduous courses, such as the Section Commander Battle Course and Pre‐Parachute Selection. The Pre‐Parachute Selection course is the physical assessment for soldiers wishing to serve with the Airborne Forces and is regarded as one of the most arduous courses in the British Army. To explain this further, all soldiers complete basic training, but to become a Paratrooper or serve with Airborne Forces, you must complete a selection process. In general, these assessments have longer load carriage distances and other arduous assessments (e.g., log run, stretcher race). I can honestly say that successful completion of these courses was possible only because of my change in body mass. From a scientific perspective, both body composition and the external load carried relative to body mass are important elements for understanding the metabolic demands and for success in a given load carriage task. For example, Lyons et al. (2005) demonstrated that expressing lean body mass relative to dead mass (fat mass plus external load mass) provided strong correlations with the metabolic demands of a 40 kg load carriage task; far stronger than lean body mass alone. At a more generic military performance level, Allison et al. (2019) demonstrated that both men and women in a cluster of higher‐performing United States Marine Corps Combat Fitness Tests had a lower fight load index [(fat mass + external load)/fat‐free mass] compared with the worst‐performing cluster. Likewise, in an anaerobic endurance military simulation test, dead mass ratio [body mass/(fat mass + external load)] was the strongest predictor of performance (Pihlainen et al., 2018).

When I left the British Army in 2021, I weighed 73 kg and still managed to complete the physical assessments easily, despite the changes outlined in Table 1. Using an arbitrary external load mass of 40 kg and using my body masses at the beginning and end of my career, this would have meant that I was carrying ∼70% of my body mass at the start of my career, compared with ∼57% when I left. Interestingly, throughout my time in the British Army, I always wondered about those older and more experienced soldiers (including myself at the end of my career) who were still able to complete the load carriage assessments with relative ease. Although there is no doubt that there is a mental component to this, I would also suggest that personal experience contributes significantly. This experience leads to improvements in areas such as how to pack equipment correctly, preparing for the assessment (taping up feet, hydration and feeding) and ensuring that boots are worn in and that personal sock preference has been found. From my experience, most soldiers seem to start finding load carriage easier around the time they reach the rank of Seargeant, which is ∼8–12 years into service (depending on role and trade). When we consider that soldiers have conducted not only load carriage over this period but a significant volume of strength training and general conditioning, their training age would probably be significantly higher than the younger cohort.

## 3 LOAD DISTRIBUTION

The way in which the load has been carried changed drastically across my time in the British Army. Traditionally, equipment was carried using belt‐type equipment with large pouches attached (collectively termed webbing) alongside bergens (large rucksacks) and day sacks (small rucksacks). The use of webbing places most of the belt kit load on the posterior of the soldier to allow soldiers to crawl along the ground without being impeded. In contrast, soldiers in more recent years have increasingly opted for a less ‘traditional’ approach of carrying a military belt kit that mostly consists of ammunition, weapon ancillaries and emergency food via a chest/front‐worn method. Typically, this involves using smaller pouches mounted to their body armour. This method has not only allowed the centre of mass of the load to straddle the centre of mass of the body, resulting in lower energy expenditure, but also reduces the amount of forward trunk lean; a mechanism linked to lower back injuries (Lloyd & Cooke, 2011). Personally, I found the torso chest rig my preferred configuration owing to feeling more upright and agile. I also found less discomfort and fatigue in my lower back during load carriage tasks. One downside of having chest‐worn equipment is the restriction of movement. For example, trying to stand up from the ground quickly is difficult with the added weight at the front of the torso. Moreover, we were aware of the risk of carrying equipment on the front of the torso, with some soldiers being injured during explosions by equipment on the front of the torso being pushed upwards. To address this, I tried where possible to use pouches with a closed top. However, some equipment might need to be used quickly in a firefight, such as a pistol or changing magazines, hence a conscious decision on this trade‐off was often required. One thing I noticed over my military career, which was echoed by others, was that when the pace during load carriage was slow, I seemed to feel the load on the shoulders more. Focusing on a faster pace to keep moving seemed to keep my mind occupied on the physical exertion as opposed to the load I was carrying.

From a physiological perspective, torso‐borne load has been demonstrated to compress the thoracic cavity, leading to inspiratory resistance and reduced pulmonary function. Notably, the increased load on the torso, as has been the ‘trend’, has been shown to increase fatigue of the respiratory muscles and increase expiratory flow limitation, when compared with lighter loads and with loads carried in a backpack (Armstrong et al., 2019; Faghy & Brown, 2019). Again, from experience I found that when the torso‐borne equipment and body armour were too tight around the torso, I could feel an increase in breathing restriction, which would make physical tasks harder to complete. It was trial and error to ensure that the equipment was as close to the body as possible to ensure safety and unwanted movement but also not to hinder my ability to breathe. However, my preference for the chest‐worn equipment allowed me to reduce the tightness of the body armour, because I found that the chest‐rig configuration helped to hold the body armour in place, increasing comfort and seemingly reducing the breathing restriction.

## 4 MUSCULAR FUNCTION

Given that load carriage is a means of transiting from one location to another, it is rarely a discrete task, but instead part of a series of tasks to attain a military objective. A good example of this is the new Physical Employment Standards for the British Army, whereby soldiers go from a load carriage assessment into a simulated fire and manoeuvre task into a casualty drag task; a design which mirrors a plausible mission scenario. An example from my own experience was during the Iraq War (2003), where we conducted a deliberate dawn attack. We patrolled ∼6 km fully loaded with ammunition and anti‐tank weapons [owing to a possible armour (tank) threat]. We arrived at the starting point and started the deliberate company attack, which lasted ∼2 h. Once the enemy position was secure, we were required to search the area and each enemy position. The whole action took ∼12 h, with the load carriage element forming only a small portion of the whole action. During this action there was little time to remove equipment. There was sometimes the ability to remove patrol sacks whilst conducting some local clearance tasks and some small periods when helmets could be removed and body armour opened to try allowing air to circulate, but this was minimal. From a physical perspective, it is therefore important to understand the likely influence of load carriage on subsequent taskings, and as a commander it is important to manage the workloads of soldiers to ensure that they arrive in a state conducive to completing their subsequent taskings. Within the scientific literature, neuromuscular performance has typically been the focus of this research area. For example, Fallowfield et al. (2012) demonstrated a decrease in jump height (8% ± 9%) and power (5% ± 5%), following a 19.3 km field‐based load carriage task (4.2 km h^−1^, carrying 31.0 kg) conducted by Royal Marine recruits. Likewise, for three repeated bouts of load carriage, we recently demonstrated a ∼25% mean reduction in peak maximal isometric voluntary contraction of the quadriceps and a ∼12% reduction in weighted countermovement jump height across measurement points (Vine et al., 2024). In both examples, these reductions would be likely to have significant implications for physical and skilled task performance of military personnel (Fallowfield et al., 2012). Interestingly, during the 20 mile assessment on Pre‐Parachute Selection, I found the pace not to be so challenging, but owing to the duration of the assessment (4 h 15 min) and the undulating ground, I still found this assessment challenging.

## 5 THERMAL

During my time in Afghanistan, patrols lasted 2–6 h, with some missions extending over several days. For instance, during Operation Eagle Summit in 2008, British soldiers were deployed for 5 days in temperatures reaching 40°C, while carrying heavy equipment and engaging in combat. From a load carriage perspective, it is well documented that clothing and personal protective equipment (e.g., body armour and helmets) can impede heat‐loss mechanisms, which can degrade performance and exacerbate the risk of heat strain (Caldwell et al., 2011; Parsons et al., 2019). From my own experiences, wearing body armour noticeably insulated the torso and hindered my ability to dissipate heat. At the time, behavioural and clothing changes were the only cooling strategies available to us, although more advanced heat‐dissipation methods appropriate for the military have been suggested (e.g., arm immersion cooling; Lee et al., 2015). Managing work rate in accordance with wet‐bulb globe temperature and ensuring proper acclimatization were also important to our management of the environmental conditions. Given the environmental conditions and our inability to carry sufficient fluids, dehydration was also a significant concern for myself and the soldiers under my command. Although the thermal physiology of military performance is a separate topic, this repercussion of load carriage should be acknowledged owing to: (1) the increases in metabolic heat production associated with the increased metabolic work rates associated with carrying additional load; and (2) the reduced ability to dissipate heat associated with load carriage equipment (e.g., bergens and body armour) worn around the torso. For this reason, tools such as the Heat Strain Decision Aid have been received considerable attention within the military thermal physiology literature, to support personnel and try to minimize heat‐related injuries (Potter et al., 2017).

## 6 SUMMARY

Although efforts to reduce the external load mass carried by soldiers have been made, the necessity to introduce and carry new technologies has not resulted in this desired outcome. Despite seeing considerable changes in load carriage assessments and our issued equipment this fundamental task has remained relatively consistent across the years. From a personal perspective, however, the physiological cost of load carriage has altered for me because of changes in load mass, task requirements and my own personal body composition. Looking to the future, the real question is, what will load carriage look like in the next 25 years? Will it include exoskeletons, human–machine teaming or a technology that has not yet come to realization? These changes could have drastic repercussions for load carriage physiology.

## AUTHOR CONTRIBUTIONS

Christopher A. J. Vine and Nicholas Schofield were responsible for the conception and design of the manuscript and for drafting and revising the work. Both authors approved the final version of the manuscript, agree to be accountable for all aspects of the work and qualify for authorship. All those who qualify for authorship are listed.

## CONFLICT OF INTEREST

The authors declare there to be no conflicts of interest/competing interests.

## FUNDING INFORMATION

None.
